# Principal Component Analysis of the Cytokine and Chemokine Response to Human Traumatic Brain Injury

**DOI:** 10.1371/journal.pone.0039677

**Published:** 2012-06-22

**Authors:** Adel Helmy, Chrystalina A. Antoniades, Mathew R. Guilfoyle, Keri L. H. Carpenter, Peter J. Hutchinson

**Affiliations:** 1 Division of Neurosurgery, Department of Clinical Neurosciences, University of Cambridge, Addenbrooke’s Hospital, Cambridge, United Kingdom; 2 Nuffield Department of Clinical Neurosciences, Department of Clinical Neurology, John Radcliffe Hospital, University of Oxford, United Kingdom; 3 Wolfson Brain Imaging Centre, Department of Clinical Neurosciences, University of Cambridge, Addenbrooke’s Hospital, Cambridge, United Kingdom; Boston University School of Medicine, United States of America

## Abstract

There is a growing realisation that neuro-inflammation plays a fundamental role in the pathology of Traumatic Brain Injury (TBI). This has led to the search for biomarkers that reflect these underlying inflammatory processes using techniques such as cerebral microdialysis. The interpretation of such biomarker data has been limited by the statistical methods used. When analysing data of this sort the multiple putative interactions between mediators need to be considered as well as the timing of production and high degree of statistical co-variance in levels of these mediators. Here we present a cytokine and chemokine dataset from human brain following human traumatic brain injury and use principal component analysis and partial least squares discriminant analysis to demonstrate the pattern of production following TBI, distinct phases of the humoral inflammatory response and the differing patterns of response in brain and in peripheral blood. This technique has the added advantage of making no assumptions about the Relative Recovery (RR) of microdialysis derived parameters. Taken together these techniques can be used in complex microdialysis datasets to summarise the data succinctly and generate hypotheses for future study.

## Introduction

Traumatic Brain Injury (TBI) is a multifaceted pathology including diverse mechanisms such as excitotoxicity, free radical formation, disrupted metabolism and brain swelling [1]. A range of cytokines and chemokines have been implicated in these pathophysiological consequences of TBI [2], [3]. Cytokines are paracrine and autocrine mediators of inflammation produced by a number of central nervous system cell types, including neurones, astrocytes and microglia and can be measured in a range of biological fluids including blood, cerebrospinal fluid and microdialysate [4]. Disentangling the roles and inter-relationships between these mediators is a key goal in developing a mechanistic understanding of neuronal loss following TBI as well as identifying novel therapeutic targets [5]. However, there are a number of practical difficulties in interpreting human cytokine data due to the large number of inter-related variables, variations in monitoring period in relation to time of injury and missing data points [6]. Many authors have attempted to use univariate correlations between a given mediator and a clinical outcome to draw inferences regarding the biological action of a cytokine, often pigeon-holing a cytokine as ‘beneficial’ or ‘damaging’ [7], [8], [9], [10], [11], [12], [13], [14], [15], [16], [17], [18]. This approach may be flawed for several reasons both in this context and within the wider TBI biomarker literature. Firstly, as there is a common trigger to cytokine production, namely TBI, the change in concentration of these mediators will likely correlate with each other and with the severity of injury irrespective of their ultimate biological action. Secondly, it is clear that cytokines that are directly antagonistic to one another at the same receptor (such as IL1β and IL1ra) are both produced in response to TBI in concert and may therefore correlate positively with each other [19]. Thirdly, the actions of a given cytokine are dependent on the time period and context in which it is produced [3], [20]. There is therefore a need for multivariate statistical techniques that incorporate the putative statistical interactions between cytokines in order to interpret and analyse cytokine data of this type. Multivariate regression is a ubiquitous technique within the biological literature, however it is limited by the need for large numbers of subjects in relation to the number of variables measured to prevent overfitting. In addition, without interpolation techniques, multivariate regression cannot compensate for missing data points potentially requiring observations to be discarded from the model. In contrast, multivariate projection methods such as principal component analysis (PCA), partial least squares discriminant analysis (PLS-DA) and their various derivatives provide a powerful method for exploring complex datasets with multiple variables and missing data points with relatively small numbers of observations [21], [22], [23].

We have previously described the measurement of 42 cytokines in brain extracellular fluid in 12 patients with severe TBI for 5 days at 6 hourly time points as well as in concurrent blood samples [6]. We demonstrated a stereotyped sequence of cytokine production within the brain following injury as well as significantly higher concentrations of some cytokines in brain compared with blood. We have investigated this dataset using multivariate projection methods in order to explore the underlying structure within the cerebral cytokine response. Firstly, we have sought to identify the clusters of cytokines that discriminate between patients as a focus for future studies into neuroinflammation following TBI. Secondly, we have explored the change in this pattern over time. Thirdly, we have sought to identify the differences between the innate inflammatory response within brain extracellular space compared with that in peripheral blood following trauma.

**Table 1:** Cytokines Analysed.

Cytokine	Abbreviation
Epidermal Growth Factor	EGF
Eotaxin	Eotaxin
Basic Fibroblast Growth Factor	FGF2
Fms-related tyrosine kinase 3 ligand	Flt3 lig
Fractalkine	Frac
Granulocyte Colony Stimulating Factor	G-CSF
Granulocyte-Monocyte Colony Stimulating Factor	GM-CSF
GRO	GRO
Interferon α-2	IFNa2
Interferon γ	IFNg
Interleukin-1 α	IL1a
Interleukin-1 β	IL1b
Interleukin-1 receptor antagonist	IL1ra
Interleukin-2	IL2
Interleukin-3	IL3
Interleukin-4	IL4
Interleukin-5	IL5
Interleukin-6	IL6
Interleukin-7	IL7
Interleukin-8	IL8
Interleukin-9	IL9
Interleukin-10	IL10
Interleukin 12 subunit β	IL12p40
Interleukin-12	IL12p70
Interleukin-13	IL13
Interleukin-15	IL15
Interleukin-17	IL17
Chemokine (C-X-C motif) ligand 10	IP10
Monocyte Chemotactic Protein 1	MCP1
Monocyte Chemotactic Protein3	MCP3
Macrophage Derived Chemoattractant	MDC
Macrophage Inflammatory Protein-1α	MIP1α
Macrophage Inflammatory Protein-1β	MIP1β
Platelet Derived Growth Factor AA	PDGF-AA
Platelet Derived Growth Factor AB/BB	PDGF-AAAB
RANTES	RANTES
Soluble CD40 Ligand	sCD40L
Soluble Interleuking-2 Receptor	sIL2R
Transforming Growth Factor α	TGFa
Tumour Necrosis Factor α	TNF
Tumour Necrosis Factor β	TNFb
Vascular Endothelial Growth Factor	VEGF

## Materials and Methods

In total, twelve patients with diffuse severe traumatic brain injury defined as a post-resuscitation Glasgow Coma Score≤8, a consistent mechanism of injury and consistent neuroimaging were monitored with cerebral microdialysis, arterial and jugular venous plasma sampling for a total of five days.

### Ethics Statement

The study protocol was reviewed and approved by the Cambridgeshire Local Research Ethics Committee (2). Written informed assent was taken from the next of kin of all patients in line with our locally agreed protocols with Cambridgeshire Local Research Ethics Committee (2).

### Microdialysis

CMA71(CMA Microdialysis AB, Solna, Sweden) high molecular weight cut-off microdialysis catheters (nominal cut-off 100 kDa) were perfused with 3.5% Human Albumin Solution made up in CMA CNS perfusion fluid) (Pharmacy Manufacturing Unit, Ipswich Hospital NHS Trust, UK), at a rate of 0.3 µl/minute, using CMA 106 (CMA Microdialysis AB, Solna, Sweden) microinfusion pumps. Samples were pooled in six hour time epochs before analysis. All catheters were placed through cranial access devices (Technicam, Newton Abbot, UK), into areas of brain that were consistent with diffuse injury on neuroimaging. At timepoints during which microdialysis was stopped for clinical indications (e.g. during MR imaging), these timepoints were noted as ‘missing’.

**Table 2:** Number of Principal Components Derived and Proportion of Variation Explained.

Model	Principal Components Derived	Variation explained (R2X)	Variation explained by first Two Principal Components (Q2X)	Figure
48 Hour Pooled Cerebral Microdialysis Data (Figures 1 and 2)				
PCA	4	63.7%	44.5%	1
PLS-DA	2	34.2%	34.2%	2
72 Hour Pooled Cerebral Microdialysis Data (Figures 3 and 4)				
PCA	3	55.6%	45.6%	3
PLS-DA	2	31.3%	31.3%	4
Plasma vs Cerebral Microdialysis Data (Figures 5 and 6)				
PCA	5	54.2%	33.4%	5
PLS-DA	3	37.4%	90.6%	6

This table provides summary data for each of the models generated in SIMCA-P+. The algorithm used continues to derive principal components until cross-validation shows that further principal components are only modelling noise within the dataset. The first 2 principal components are used in all the figures and subsequent analysis. PCA; Principal Component Analysis. PLS-DA; Partial Least Squares Discriminant Analysis.

### Plasma Sampling

Whole blood samples were taken from each patient twice daily into EDTA vials from an arterial line in the radial artery. Samples were centrifuged at 5000 rpm at 4°C for 15 minutes and the resulting supernatant aliquoted and stored at −80°C until assayed. The timing of plasma samples was also related to the time of injury as for microdialysate.

### Cytokine Analysis

The samples were analysed in duplicate using the Milliplex™ Multi-Analyte Profiling Human Cytokine/Chemokine 42 analyte premixed kit (Millipore Corp, Missouri, USA) on the Luminex 200 system (Luminex Corporation, Austin, TX, USA) running STarstation software(Applied Cytometry Systems, Sheffield, UK). Table 1 lists the cytokines and chemokines assayed. Cytokine standards were run on each plate and used to determine an eight-point five-parameter logistic standard curve. Plasma and micodialysate samples were assayed on separate plates with appropriate standards and background wells.

**Figure 1:**
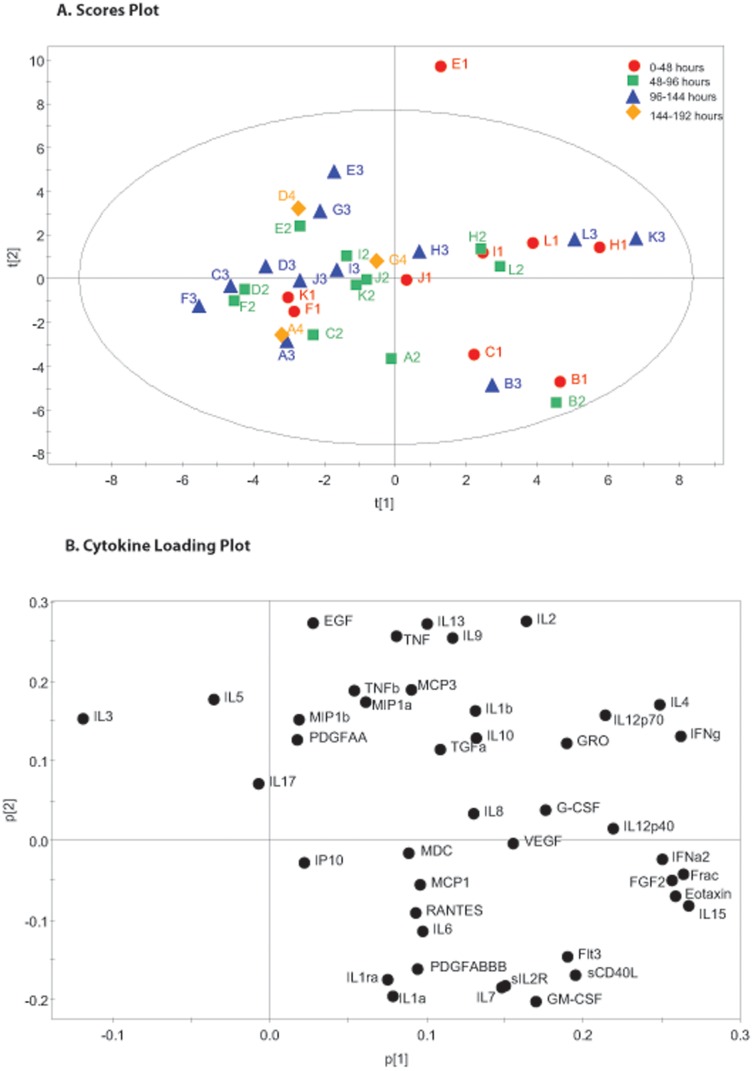
48 Hour Time Pools Principal Component Analysis. The figure shows cerebral microdialysis derived cytokine data from 12 patients (A–L) pooled into 48 hour time epochs(1–4). Principal component analysis has been used to identify the first 2 principal components which explain 63.7% of the variation in the dataset. Part A is a scores plot which shows the scores on each principal component for each of the observations. Part B is a loading plot which shows the cytokines which load on the respective principal components. Functionally related cytokines, such as IL1b and TNF, cluster within the same quadrant of the plot suggesting that they co-vary.

**Figure 2:**
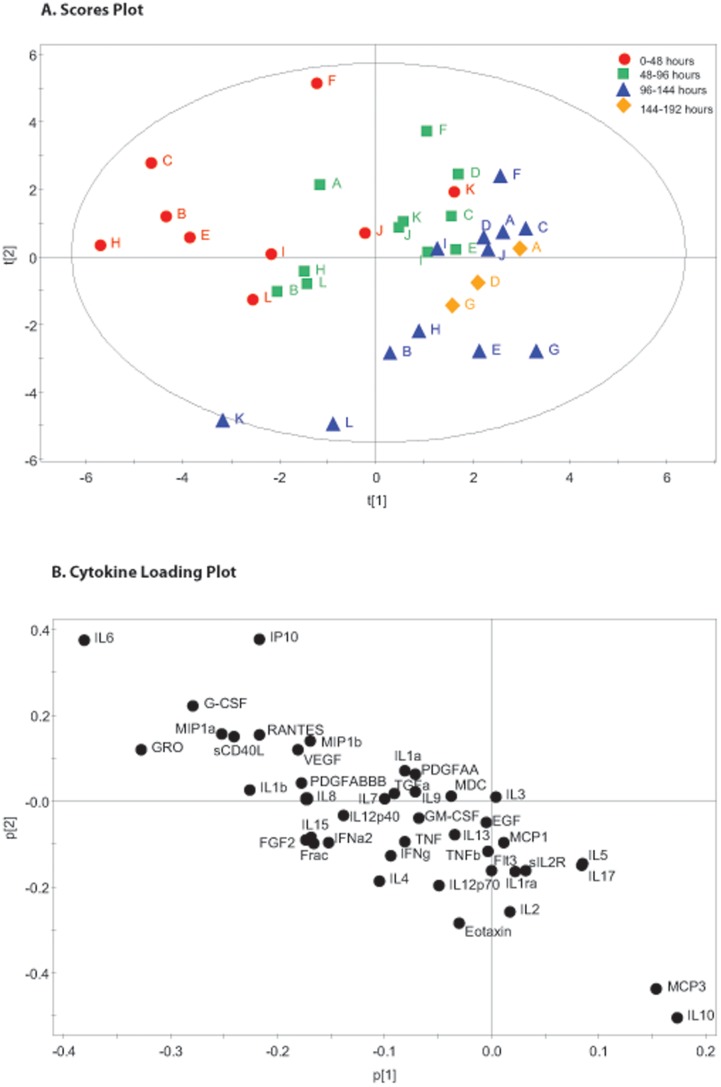
48 Hour Time Pools Partial Least Squares Discriminant Analysis. The figure shows the same cerebral microdialysis derived cytokine data as in figure 1, from 12 patients (A–L) pooled into 48 hour time epochs (Red 0–48 hours, Green 48–96 hours, Blue 96–144 hours, Yellow 144–192 hours). Partial Least Squares Discriminant Analysis is a regression extension of Principal Component Analysis in which the model identifies the greatest sources of variation between pre-specified groups of observations. In this case the supervising variable is time. There is a clear shift in the pattern of observations in the scores plot (part A) over time from the 0–48 hour epoch (red) to the 48–96 hour time epoch (green) to the later time points (blue and yellow). The loading plot (part B) illustrates the cytokines that are responsible for the pattern apparent in part a.

**Figure 3:**
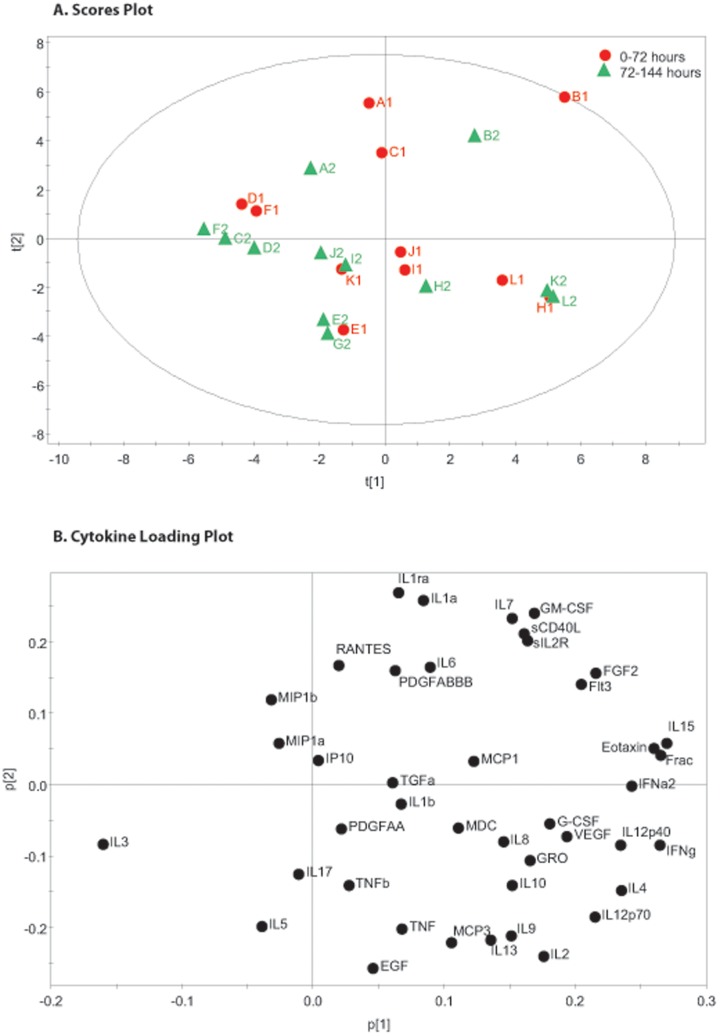
72 Hour Time Pools Principal Component Analysis. The figure shows cerebral microdialysis derived cytokine data from 12 patients (A–L) pooled into 72 hour time epochs(1,2). Principal component analysis has been used to identify the first 2 principal components which explain 55.6% of the variation in the dataset. Part A is a scores plot which shows the scores on each principal component for each of the observations. Part B is a loading plot which shows the cytokines which load on the respective principal components. In a similar pattern to figure 1, IL1b and TNF still cluster within the same quadrant of the plot.

**Figure 4:**
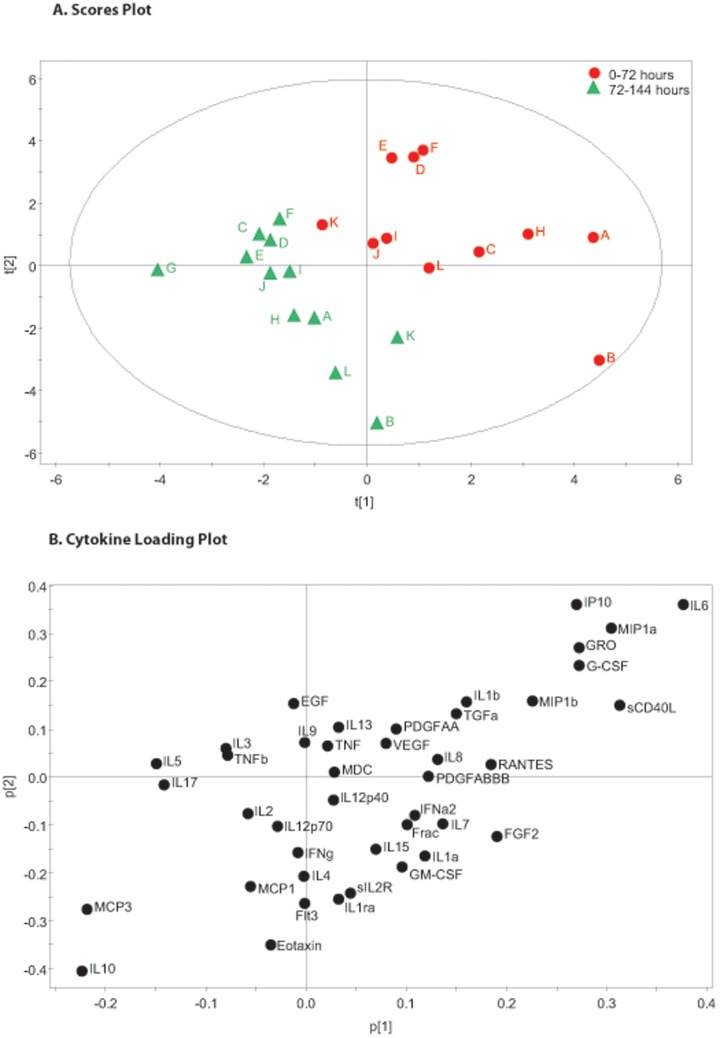
72 Hour Time Pools Partial Least Squares Discriminant Analysis. The figure shows the same cerebral microdialysis derived cytokine data as in figure 3, from 12 patients (A–L) pooled into 72 hour time epochs (Red 0–72 hours, Green 72–144 hours). Partial Least Squares Discriminant Analysis is a regression extension of Principal Component Analysis in which the model identifies the greatest sources of variation between pre-specified groups of observations. In this case the supervising variable is time. There is a clear shift in the pattern of observations in the scores plot (part A) over time from the early time points (red) to the later time points (green). The loading plot (part B) illustrates the cytokines that are responsible for the pattern apparent in part a.

**Figure 5:**
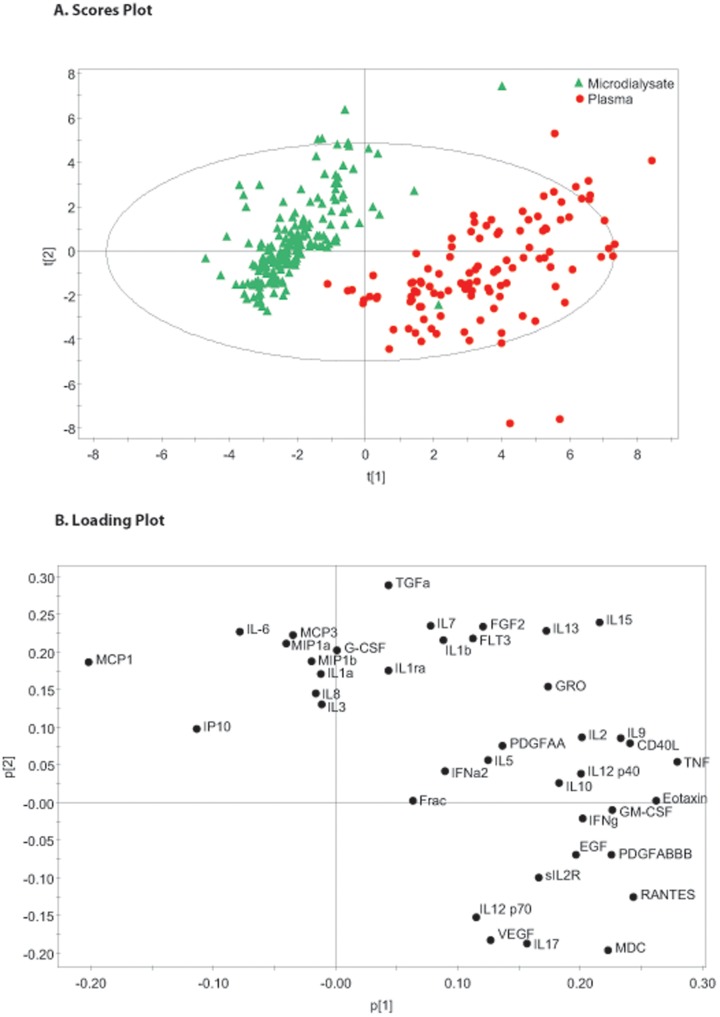
Paired Microdialysis and Plasma Samples Principal Component Analysis. The figure shows paired microdialysate and plasma derived cytokine data from 12 patients. Principal component analysis has been used to identify the first 2 principal components which explain 33.4% of the variation within the dataset. Part A is a scores plot which shows the scores for each observation on each of the principal components and even in this unsupervised model a clear separation between microdialysis and plasma derived cytokines is apparent. Part B is a loading plot which shows the cytokines responsible for the differences between the two biological compartments.

**Figure 6:**
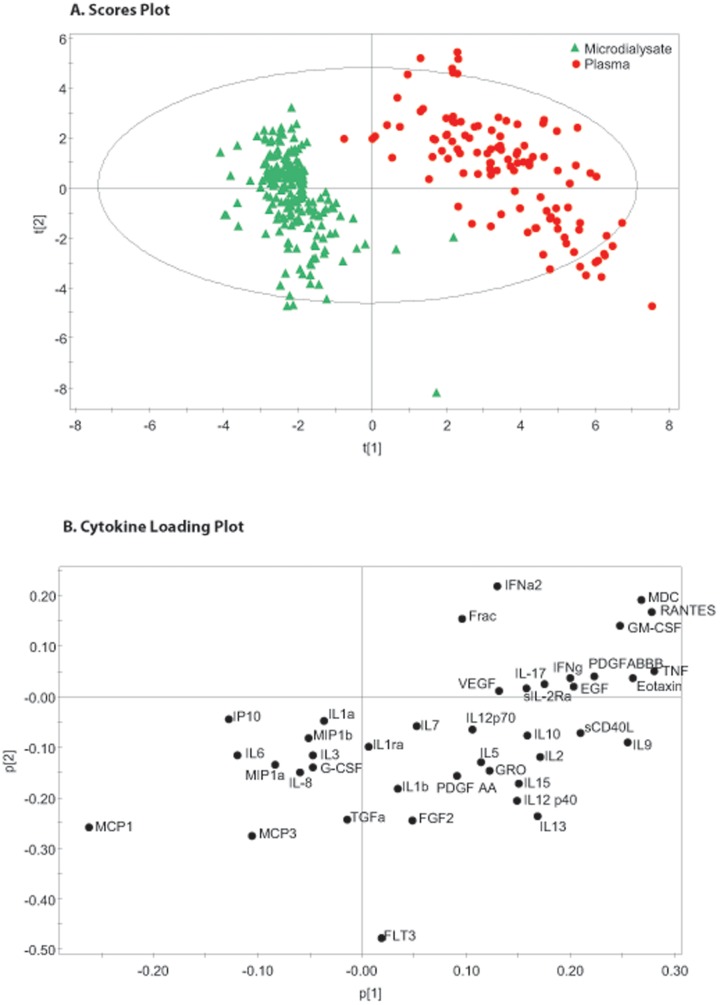
Paired Microdialysis and Plasma Samples Partial Least Squares Discriminant Analysis. This figure shows paired microdialysate and plasma derived cytokine data from 12 patients as in figure 5. In this instance the source of the sample (microdialysate vs plasma) has been used as a supervising variable within a partial least squares discriminant analysis to identify two principal components that maximise the differentiation between the two biological compartments. Part A is a scores plot which shows the scores on each principal component for each observation. As with figure 5, there is a clear separation between the two biological compartments. Part B is a loading plot which demonstrates which cytokines are responsible for the difference apparent in part a. The cytokines on the left side of the figure show a comparative neural tropism when the two biological compartments are compared.

### Multivariate Statistical Analysis

Statistical analysis was performed using multivariate techniques, specifically principal component analysis (PCA), followed by partial least squares discriminant analysis (PLS – DA). SIMCA – P+ version 12 (Umetrics, Umea, Sweden) was used to identify principal components which accounted for the majority of the variation within the dataset. PCA is an unsupervised method and a data reduction technique that allows the major sources of variation in a multi-dimensional dataset to be analysed without introducing inherent bias. PLS – DA is a regression extension of the principal component analysis that uses class information to maximize the separation between various groups of observations. To estimate the number of PCA and PLS-DA components, cross-validation was used [21]. Data for each cytokine was mean centred and variance scaled to unit variance. SIMCA-P+ uses NIPALS (non-linear iterative partial least squares) algorithm to calculate the first few principal components and inherently compensates for missing data values. This has been suggested as a more accurate, though computationally more complex method for deriving eigenvalues [24]. Cross validation was carried out by dividing the data into seven parts and comparing models with each of the seven parts left in or out in turn. Predicted Residual Sum of Squares are calculated for the whole dataset and scaled to provide the Q2 statistic.

Cerebral cytokine data was pooled into 48 hour and 72 hour bins, following the time of injury, in order to ease the interpretation of the numerous time points and minimise missing data values. The 48 hour bins are 1(0–48 hours), 2 (48–96 hours), 3 (96–144 hours), 4 (144–192 hours) and 72 hour bins are 1 (0–72 hours), 2 (72–144 hours. For transparency we have included the PCA and temporally defined PLS-DA analyses for both 48 hour and 72 hour time bins. The monitoring period for each patient in relation to injury varies, therefore data is not available for every patient for every time point.

The analysis was performed in two stages. Firstly a PCA analysis was performed to examine any intrinsic variation in the cytokine data set and whether any clustering was presented at this stage. Removal of outliers made no difference in the model and outliers are included in all models presented. Secondly, PLS – DA was performed using the cytokine data, to demonstrate the separation of the time points and also identify the cytokines responsible for any of the clustering/separation present.

Cerebral cytokine data was also compared to concurrent plasma samples using the both PCA and PLS-DA methodology. In this case, we have not sought to make a comparison across time, so individual observations (‘microdialysis’ vs ‘plasma’) have been entered into the model directly. In this case, our aim was to define a subset of neurotropic cytokines.

## Results

Six models were derived in total: the number of principal components derived, and the variation explained by these components for each of the models is summarised in Table 2. R2 describes the goodness of fit fraction of the sum of squares of all the variables explained by a principal component and Q2 describes the goodness of prediction of the fraction of the total variation of the variables that can be predicted by a principal component using cross-validation methods.

Each of the figures 1, 2, 3, 4, 5, 6 relates to one of the six models in Table 2 and is made up of two parts which are closely related. Part ‘a’ of each figure shows the scores plot obtained using SIMCA-P+. The scores plot shows the scores for each observation on Principal Component (PC)1 and PC2. Strictly speaking the SIMCA algorithm provides an estimate of the PC, which is more correctly referred to on the figures as t [1]/t [2] on the scores plots and p [1]/p [2] on the loading plots. PC is used within the text for ease of reference although we accept that they are not identical to principal components. The ellipse on the plot (Hotelling ellipse) is the 95% confidence interval for the model, hence plots of this type are sometimes referred to as Hotelling plots. Part ‘b’ in each of the figures shows the corresponding loading plot. The loading plot illustrates the relative contribution of each of the cytokines (referred to as the ‘loading’) to the two principal components. It is a linear contribution of each of these loadings that determines the score for each observation in the scores plot.

The scores plot is used to determine if there is clustering of observations, which would suggest a common underlying multivariate signal (or in this case pattern of cytokines) exists for that particular cluster of observations. Observations on the scores plot that lie within the Hotelling ellipse are within the 95% confidence interval for the model and are therefore well modelled in statistical terms. In order to interpret the cytokines responsible for a given multivariate signal reference is made to the loading plot. Cytokines with a larger loading on a PC in a particular direction contribute more heavily to the multivariate signal in that direction and therefore make a greater contribution to observations with high scores on that PC. Moreover, cytokines which cluster on the loading plot have a high degree of co-variance when the sources of variation in the model are considered.

### Principal Component Analysis and PLS – DA of Pooled Microdialysate Data

The 48 hour microdialysate pooled data for each patient is plotted individually in figure 1. The model generated four principle components that explain 63.7% of the variation within the dataset. The first two principal components are presented and together explain 44.5% of the variation within the dataset. All data points, other than E1 fall within the 0.95 Hotelling ellipse. The corresponding loading plot (Figure 1b) demonstrates distinct clusters of cytokines. PLS-DA of the same data using the time point as the supervising variable (Figure 2a) demonstrates that there is a consistent shift across time in the pattern of cytokine expression following TBI. The corresponding PLS-DA plot (Figure 2b) illustrates the cytokine loadings responsible for this shift. The same analysis was repeated on 72 hour time bins and is presented in figures 3 and 4. The same features are apparent within these models despite the difference in time epoch used. Namely, the observations fall within the Hotelling ellipse, distinct clusters of cytokines are apparent in the PCA loading plot (Figure 3b), PLS-DA demonstrates a clear separation of observations (Figure 4a) and the same cytokines are responsible for this multivariate signal on the PLS-DA loading plot (Figure 4b) as in the 48 hour time epoch (Figure 2b).

### Comparison between Cerebral and Plasma Cytokine Profiles

We have also performed a PCA on the data set of paired blood and MD data to determine whether a multivariate signal was present. Figure 5 shows a PCA of this data demonstrating clear separation in cytokine profiles even within the unsupervised model. Figure 6 shows the corresponding PLS-DA data reinforcing the separation in cytokine profile between these two biological compartments. The cytokines appearing to the left of the PLS-DA scores plot (Figure 6b) are more negatively loaded on PC1 and seem to be responsible for the separation in patterns of response between brain extracellular space and plasma.

## Discussion

### Principal Component Analysis of Microdialysate Data

PCA is an unsupervised dimension reduction technique which generates latent variables designated *prinicipal components.* The first PC is a linear combination of each of the original variables which incorporates the greatest sources of variation within a dataset. The second and subsequent PCs are further latent variables that explain the greatest sources of variation that are left over beyond the first PC and lie orthogonal to it. In our initial analysis we have incorporated data from all 12 patients for a range of time-points pooled into 48 and 72 hour bins. Exploring the sources of variation within this dataset using PCA gives an indication of the cytokines responsible for variation between patients and over time, without making any assumptions about which patient or which time-point each observation has come from. Each observation is therefore made up of 42 individual cytokine concentrations from a given patient (A–L) at a given time-point (1–4 in the 48 hour pools and 1–2 in the 72 hour pools). Comparing the models generated using 48 hour and 72 hour time pools demonstrates clustering of the same groups of cytokines. This suggests that the models are robust and do not simply reflect idiosyncrasies of the time pools presented. The polarity of some of the PCs are inverted when figures 1 and 2 are compared with figures 3 and 4, however this does not change the interpretation of the plots and is a reflection of the algorithm used to generate the PC. Table 2 lists the amount of variation explained by each of the models demonstrating that for both the 48 hour and 72 hour pools ∼45% of the total variation in the dataset is explained by just the first two PC. Given the recognised heterogeneity in human TBI [25], this degree of dimension reduction whilst explaining a large degree of variation adds credence to the robustness of the models.

The loading plots (Figure 1b, Figure 3b) plot the relative contribution of each cytokine to the respective PC illustrated in in the scores plots. The loading plots allow us to explore the largest sources of variation within the dataset and identify clusters of cytokines that closely co-vary. The current literature on TBI and cytokines focuses on a relatively small group of mediators. The two cytokines most commonly implicated in a pro-inflammatory role are IL1β and TNF. These cytokines share intracellular transduction mechanisms [26] and have synergistic actions in cell culture models [27], [28]_ENREF_35. They appear in the same quadrant in both PCA loading plots (top right figure 1b, bottom right figure 3b) suggesting concomitant production. Conversely, IL1ra, an endogenous competitive antagonist to IL1β appears in the opposing quadrant of the loading plots (bottom right figure 1b, top right figure 3b). Interestingly, IL1α, another agonist at the IL-1 receptor appears immediately adjacent to IL1ra and therefore closely co-varies with IL1ra. This relationship has not previously been described in the literature. It is not possible to determine what the functional consequences of this relationship are simply based on the PCA model, however it may be that as IL1α is produced concurrently with IL1ra it may not act as effectively as an agonist at the IL1 receptor as IL1β. There is pre-clinical evidence demonstrating differential effects of IL1α and IL1β [29] as well as a more prominent role for IL1β in inducing neurodegeneration than for IL1α [30]. This potentially adds to the considerable complexity in the regulation of the IL1 receptor pathway [31] and may result in differences in the potencies of IL1α and IL1β action *in vivo*. Furthermore, it clearly illustrates how biological mediators with opposing functions can closely co-vary. There is no way to definitively infer what a PC represents biologically, however, on the basis of the location of TNF, IL1β and IL1ra and the prior evidence for the role of these cytokines following TBI, we would suggest that PC2 represents some aspect of the pro-inflammatory consequences of IL1β/TNF action in contrast to the cytokines loading negatively on this axis.

Several chemokines were recovered using microdialysis and the loading plot reveals that many of these are produced concomitantly. For example, MIP1α (CCL-3) and MIP1β (CCL-4) appear adjacent to each other and can both signal through the CCR5 receptor [32]. The functional consequences of this relationship are not known and have never been investigated directly in the context of neuro-inflammation. In this way the PCA model can generate hypotheses relating to specific mediators that are produced in concert *in vivo*.

The relationship between humoral and cellular inflammation has been explored extensively in the peripheral immune system. There is increasing interest in the role of microglia following TBI and their ability to carry out a functional switch between a pro-inflammatory and a reparative role. The nomenclature used by different authors varies, however IFNγ is thought to promote a pro-inflammatory phenotype (classical-activation/Th1 type response/M1 subtype) while IL-4 promotes an anti-inflammatory response (regulatory/Th2 type response/M2 subtype). IL4 and IFNγ, appear immediately adjacent to each other on the far right of the loading plot. Furthermore, both IL10 and IL12 have been implicated in phenotypic plasticity of macrophages and they also appear within in the same region of the loading plot [33], [34].

### Partial Least Squares Discriminant Analysis Using Pooled Timepoints

As well as looking for sources of variation in the dataset as a whole, multivariate projection techniques can also look for sources of variation between pre-specified observations. These so-called supervised techniques include PLS-DA. In this case, we have chosen to define ‘time following injury’ as the supervising variable. This allows an identification of the underlying patterns of cytokine that are responsible for changes in time in this patient group. The degree of variation explained by the models necessarily drops to around 1/3 of the total variation (Table 2) as the PCs now maximise variation specifically within in the time domain.

Figure 2a demonstrates that there is separation of the time points on the scores plot with observations at the first time point clustering towards the top left quadrant and observations from the third time point clustering towards the lower right quadrant. Figure 4a shows even clearer separation of the two time points, although the polarity of the PC is inverted. This pattern is not replicated for every patient (e.g. patient K moves from top right quadrant towards the bottom left quadrant in figure 3a and from top right quadrant to bottom left quadrant in figure 4a) however the observations fall within the Hotelling 95% significance ellipse, again suggesting that the PLS-DA model is an accurate representation of the underlying dataset. It is not clear what is responsible for this difference but no inference can be made solely on the results from a single patient.

Inspection of the loading plots for the PLS-DA (figures 2b and 4b) reveal the cytokines that are responsible for the changing pattern of response over time. As the loading of a cytokine on a given PC is linearly related to the score that observation receives in the scores plot, we can infer that the cluster of cytokines in the quadrant of the loading plot adjacent to the first time point (e.g. IL6, GRO, G-CSF, IP10) are produced earlier following injury in relation to cytokines appearing in the opposite quadrant (e.g IL10, MCP3, IL17). These models therefore suggest that there are distinct temporal phases to the innate inflammatory response to brain trauma. An important caveat to the interpretation of the cytokines responsible for ‘early’ vs ‘late’ patterns, is that microdialysis monitoring in patients is not available immediately at the time of injury. In practice, a minimum of 24 hours elapses before a patient is resuscitated, transferred to neurocritical care and monitoring is instituted. For this reason, cytokines that are known to be produced and released within the first 24 hours following injury are likely to be under-represented within this model as their levels are already likely to have peaked and may have returned to baseline levels at the time of monitoring. We would therefore expect that cytokines that peak at 24–48 or 24–72 hours are most likely to load highly for the early time points. From an analysis of the time at which each cytokine has its highest (peak) value following TBI [6], this would appear to be the case for IL6, G-CSF and IP10. Similarly IL10 appears to have its highest values at day 5–6 and appears in the later time points. Both, IL10 and IL17 have both been implicated in the interaction between cells of the macrophage lineage and regulatory T-cell responses [33], [35]. This temporal shift may therefore represent a shift from innate to adaptive immunity in the cerebral cytokine profile following TBI.

### Comparison between Microdialysate and Plasma Patterns of Cytokine Production

We have also explored the relationship between the systemic inflammatory response, as gauged within plasma, and the cerebral inflammatory response to trauma. In order to make this comparison we have used temporally paired serum and microdialysate samples assayed using the same technique for the same substances. Figure 5a shows the hotelling plot and loading plot for the entire dataset. It is immediately apparent that even in an unsupervised model, there is a clear separation between observations made in the two compartments. We have also carried out a PLS-DA on this dataset using the biological source of the sample (microdialysate vs plasma) as the supervising variable (Figure 6). The close concordance between the loading plots in the PCA and PLS-DA models suggests a robust differentiation between the two compartments.

The individual mediators involved in inflammation are ubiquitous and implicated in several contexts and pathologies. Identifying tissue specific variations provides an insight into the subtleties of the inflammatory response in TBI. Almost all the microdialysate observations load negatively on PC1 in contrast to the plasma samples (Figure 6). The cytokines loading most heavily in this direction are the chemokines MCP-1, MCP-3, MIP1α, MIP1β, IP-10 and the cytokines IL-6 and IL-8. Several recent studies have highlighted the importance of MCP-1 (CCL-2) in the pathogenesis of TBI in animal models [36], [37]. It has also been shown to modulate cytokine production in a mouse culture model [38]. This comparative neural tropism of this group of chemokines suggests that, in response to TBI, they are playing a more pronounced role centrally rather than systemically. This is not to say that the other mediators are unimportant in TBI, however the patterns of expression are more equally distributed between the central and peripheral compartments. The same caveats regarding monitoring period also apply in this context i.e. cytokines and chemokines that may show differences between the two biological compartments in the first 24 hours following injury will not be identified in this analysis.

### Advantages of Multivariate Projection Methods

There is a growing recognition that humoral mediators of innate inflammation (i.e. cytokines and chemokines) play a mechanistic role in the pathophysiological processes in a range of neurological disorders including HIV encephalitis [39], ischaemic stroke [40] as well as in TBI [3]. Microdialysis is unique in its ability to sample the extracellular fraction of soluble mediators, providing a direct and temporally distinct proxy for the biology of the brain extracellular space [41]. However, biomarker studies of this type are intrinsically limited in their interpretation by the fact that each of the measured variables is likely to show multi-collinearity with other related variables. Studies which sample only a few or even single cytokines within a biological compartment and attempt a univariate correlation with clinical parameters or with other biomarkers may be confounded by a range of other factors such as severity of injury or other unmeasured biomarkers [5], [42]. This can lead to erroneous conclusions about the role of cytokines as beneficial or harmful based on these simplistic correlations. As our understanding of innate inflammation following TBI has developed, it has become apparent that a given cytokine may play a dual role, damaging or reparative, depending on the context in which it is expressed either in terms of timescale or the co-existent cytokine milieu [3], [5], [43], [44], [45]. The complexity and subtleties of these interactions are difficult to model mathematically, however, without taking these putative interactions into account an understanding of cytokine biology will continue to elude us. Multivariate regression has been used extensively in the TBI literature to relate a range of variables to clinical outcome following TBI including clinical parameters (such as age, GCS, pupillary function) [46], [47], [48], microdialysate parameters (L/P ratio) [49] and serum parameters (Hb, PT) [50]. This statistical method requires large numbers of observations as the number of variables increases and often take measures at single timepoints (e.g. admission serum parameters) [50] or take a mean of the measured variable (e.g. L/P ratio over monitoring period) [49] to reduce the number of variables incorporated to a manageable number [51]. This has the potential to discard potentially useful temporal information. Cytokine data, in particular, is characterised by marked rises in concentration that are short lived. If the concentration of a cytokine is averaged over the entire monitoring period (equivalent to the area under the time-concentration curve if sampling time points are evenly spaced), the mean may be heavily influenced by the length of the monitoring period at which baseline levels are measured [5].

In order to address some of these issues we have utilised well characterised multivariate projection techniques, one of a range of chemometric methods [52], to explore the sources of variation within a complex human cytokine dataset. The greatest advantage of the approach presented here is that no prior assumptions are made as to which cytokines or mediators are of ‘importance’. The option to carry out multiple t-tests comparing a subset cytokines which are already well characterised, as in several of the studies referenced above, incorporates inherent bias into any analysis and perpetuates this bias in the literature as a whole. While it is impossible to draw any direct inference with regards to the biological function of a given mediator based on the analyses presented here, this does not detract from the ability to generate hypotheses and identify previously unrecognised relationships. The same statistical methodology may have additional utility in the analysis of data from alternative analytical techniques in TBI such as proteomics [53].

### Limitations

Microdialysis is intrinsically a focal monitor and the question arises as to whether the volume of brain sampled by the catheter is truly representative of the brain as a whole, particularly in view of the heterogeneity of TBI such that the volume sampled may be more or less injured compared with other brain areas. There is some evidence that the inflammatory response is distributed within both hemispheres, even in focal injuries [54], and patients within this study were prospectively selected on the basis of diffuse brain injuries. Another issue that has drawn attention within the microdialysis literature is that of relative recovery (RR), i.e. the proportion of a substance within the extracellular space that crosses the microdialysis membrane and can be recovered in the microdialysis fluid [55]. We have previously demonstrated that RR varies between cytokines depending on their physico-chemical properties such as pI and molecular weight [56]. For this reason the relative concentrations of mediators assayed within the microdialysis fluid may not reflect the absolute concentrations within the brain extracellular space. However, the multivariate projection methods employed compensate for both differences in RR and for variations in the underlying degree of injury sampled by the catheter, by normalising the data. In this way the model identifies **patterns** of response between mediators unrelated to the absolute concentration of any given mediator in any given catheter. This is one of the key advantages of these techniques in analysing microdialysis data. This normalisation also allows for a direct comparison in the patterns of expression between the responses in plasma and brain extracellular space irrespective of absolute values. While an estimate of RR is not required to carry out these analyses, comparisons between patients still require the RR to be consistent between catheters and patients. The factors affecting RR have been reviewed extensively in the literature [55], [56]. Mediators that have a high random variation in RR are less likely to be contribute to variation in supervised models, such as the PLS-DA, and will therefore have a diminished loading on a given PC.

A further criticism of microdialysis for assessing the inflammatory response to trauma is the suggestion that insertion of the catheter, in itself, triggers an injurious response that is superimposed on the response to the initial trauma. The existing cerebral microdialysis literature provides evidence against this [5]. Firstly, cytokines that are shown in animal models to be produced early (e.g. IL1β, TNF) following injury are not sampled in patients in which monitoring commences later following injury [6]. If the cytokine response was in response to catheter insertion, there would be a consistent temporal response irrespective of the time of catheter insertion to time of injury. Secondly, there are pathology specific differences in production of cytokines when conditions such as subarachnoid haemorrhage [57] and tumour [58] patients are compared with those following TBI [6]. Thirdly, there appears to be a stereotyped sequence of cytokine production when the time lag between the time of injury and the ‘peak’ cytokine concentration is observed [6]. This is reinforced in this study in Figures 2 and 4. The pattern of cytokine response is apparent despite the variation between patients in the time frame over which monitoring occurs. The clustering of time points occurs when the time following injury is used rather than time from monitoring. It can be seen in this figure that some patients did not have any samples in the first 48 hours, however their pattern of cytokine production remains consistent with patients that did have early monitoring. It should be pointed out that the issue of ‘trauma artefact’ is a contentious one in the microdialysis literature [59] and some authors have suggested discarding the initial samples (e.g. the first 24 hours [60]) following catheter insertion despite the points made above.

In the analysis of cerebral cytokines we have chosen to pool our data into 48 and 72 hour blocks. There is a balance to be struck between smoothing out random variations at a given time-point and maintaining an appropriate degree of temporal resolution. Taking a mean over a period of time also helps to minimise missing data points and adds a degree of clarity to the scores plots in figures 1, 2, 3, 4. We accept that this is entirely subjective and is in the authors’ opinion the time frame which provides the clearest representation of the data.

All the models presented are derived from the same cohort of 12 patients. There is a risk that the models presented are an idiosyncrasy of the particular dataset used to generate them. In statistical terms this is referred to as ‘over-fitting’. Cross-validation is used to provide an estimate of the predictive ability of the model however ultimately the most stringent test would be to collect data from a further cohort of patients and plot them on the same PCs to provide an empirical validation.

One issue that we have not been able to address is the reasons for any variation in cytokine pattern seen between patients. It would be tempting to pick out other clinical factors such as GCS or an outcome measure such as Glasgow Outcome Score (GOS) and attempt a PLS-DA. However, in a small cohort of patients such as this, we do not feel this would be scientifically justifiable. These analyses will require larger patient numbers in order to utilise multivariate techniques to analyse clinical parameters such as these. Ultimately, the most useful application of PLS-DA may be in interventional studies in which patient populations that are subjected to differing treatment paradigms can be compared in terms of their biological response. This may provide a more sensitive chemical surrogate for treatment efficacy than conventional outcome measures such as GOS.

Microdialysis has been evaluated extensively as a monitor for cerebral metabolism following TBI [49], [61] however our understanding of the biology of cytokines and chemokines in this context is not at a stage where we can promote its use as a clinical tool. Furthermore, the time and cost of recovering these samples, laboratory analysis and statistical testing does not lend itself to ‘on-line’ clinical decision making.

### Conclusion

In this study we have utilised multivariate projection techniques to reveal how inflammatory mediators demonstrate a distinct pattern of response to TBI in humans. Firstly, we have shown that several mediators show close co-variance (e.g. IL1α and IL1ra) indicating that they are produced in concert as a result of injury. Secondly, we have identified cytokines and chemokines that are produced at defined time points (e.g. IL6 at 24–48 hours, IL-10 at 96–144 hours) and discriminate between different temporal phases of the inflammatory response. Lastly, we have demonstrated that there are tissue specific variations (brain vs blood) in the patterns of mediators that are produced as a result of TBI.

In an observational study such as this no inference can be made as to the specific functions attributable to a given cluster of mediators, however by empirically determining the patterns of response the interactions of specific mediators can be explored further in animal and cell culture models. In particular, these techniques compensate for the inherent difficulties related to analysing multiple closely related mediators related to multi-collinearity, missing data points, mediators produced at different absolute concentration ranges and data from small numbers of patients. We envisage that this method can be extended into randomised studies in which cytokine data can be compared between patients receiving specific interventions.
